# Conditional Facilitation of an Aphid Vector, *Acyrthosiphon pisum*, by the Plant Pathogen, *Pea Enation Mosaic Virus*

**DOI:** 10.1673/031.010.14115

**Published:** 2010-09-15

**Authors:** Simon Hodge, Glen Powell

**Affiliations:** Division of Biology, Imperial College London, SW7 2AZ, UK

**Keywords:** alate, growth rate, *Pisum sativum*, plant virus

## Abstract

Plant pathogens can induce symptoms that affect the performance of insect herbivores utilizing the same host plant. Previous studies examining the effects of infection of tic bean, *Vicia faba* L. (Fabales: Fabaceae), by *pea enation mosaic virus* (PEMV), an important disease of legume crops, indicated there were no changes in the growth and reproductive rate of its primary vector the pea aphid, *Acyrthosiphon pisum* (Harris) (Hemiptera: Aphididae). Here, we report the results of laboratory experiments investigating how *A. pisum* responded to PEMV infection of a different host plant, *Pisum sativum* L., at different stages of symptom development. Aphid growth rate was negatively related to the age of the host plant, but when they were introduced onto older plants with well-developed PEMV symptoms they exhibited a higher growth rate compared to those developing on uninfected plants of the same age. In choice tests using leaf discs *A. pisum* showed a strong preference for discs from PEMV-infected peas, probably in response to visual cues from the yellowed and mottled infected leaves. When adults were crowded onto leaves using clip-cages they produced more winged progeny on PEMV-infected plants. The results indicate that PEMV produces symptoms in the host plant that can enhance the performance of *A. pisum* as a vector, modify the production of winged progeny and affect their spatial distribution. The findings provide further evidence that some insect vector/plant pathogen interactions could be regarded as mutualistic rather than commensal when certain conditions regarding the age, stage of infection and species of host plant are met.

## Introduction

Plant pathogens and insect herbivores can interact when they co-exist on the same host plant: they might compete directly for plant resources or interact indirectly via induced changes in plant morphology, physiology and the activation of plant defences (Hammond and Hardy 1988; Apriyanto and Potter 1990; Barbosa 1991; Stout et al. 2006; Jiu et al. 2007). These ‘tripartite’ plant-insect-virus interactions are further complicated when the pathogen is obligately dependent on the insect for its transmission. The overall interaction between the pair of species is now a combination of facilitation of the pathogen by the vector and the varying reciprocal response in the insect, and can lie anywhere along a continuum between mutualism (+, +), commensal (+, 0) and contramensal (+, -) (Hodge and Arthur 1996). It can be envisaged that there would be evolutionary pressures on the pathogen not to be antagonostic towards its insect vector and that those pathogens that modified plant biology so as to improve vector performance would subsequently be more successful in terms of their own transmission (Blua and Perring 1992a; Power 1992; Eliot et al. 2003; Maris et al. 2004; Belliure et al. 2005).

Various estimates suggest that aphids account for the transmission of between 25–50% of the plant viruses disseminated by insects (Nault 1997; Ng and Perry 2004; Hogenhout et al. 2008). A number of previous field and laboratory investigations have examined the responses of aphids to infected host plants (see reviews in Hammond and Hardy 1988; Stout et al. 2006). Aphids developing on virus-infected plants have been demonstrated to show reduced, improved or no change in individual and/or population growth rates on infected plants, depending on the system examined (Hammond and Hardy 1988; Castle and Berger 1993; Stout et al. 2006; Donaldson and Gratton 2007). It is often found that the distribution of aphids exhibits a bias towards virus-infected plants (Macias and Mink 1969; Eckel and Lampert 1996; Fereres et al. 1999) although this is not always the case (see Castle et al. 1998). There are also reports of increased production of winged alate-form progeny on infected plants, a factor liable to enhance subsequent dispersal of the plant pathogen (e.g. Gildow 1980; Blua and Perring 1992b; Fiebig et al. 2004; but see Hodge and Powell 2008).

*Pea enation mosaic virus* (PEMV) is a widespread aphid-borne virus that infects a number of leguminous plants, causing stunting and deformation of the plant and mottling and curling of leaves, and the disease can result in severe crop losses (c. 50%) in beans and peas (Hull, 1981; de Zoeten and Skaf, 2001). PEMV consists of a symbiotic mutualism between an *Enamovirus* and *Umbravirus* and is transmitted by a number of aphid species in a circulative persistent (non-propagative) manner. The virus can be acquired during access feeding periods of only a few minutes, and after a latent period the aphids can inoculate new plants in bouts of stylet probing less than 30 seconds duration (Hull 1981; de Zoeten and Skaf 2001; Powell 2005).

The pea aphid, *Acyrthosiphon pisum,* is responsible for the transmission of a number of viruses affecting legume field crops, including PEMV (Hull 1981; de Zoeten and Skaf 2001). *A. pisum* has previously been found to show varying responses to single and multiple virus infections of clovers, the response often being dependent upon the stage of infection and severity of disease symptoms (Marrkula and Laurema 1964; Ellsbury et al. 1985). Previously, we examined the response of *A. pisum* to PEMV infection of *Vicia faba* L. and found that although the *A. pisum* showed clear preferences for settling on the yellow foliage of virus-infected plants there were no effects on their growth, reproductive output or production of winged progeny (Hodge and Powell 2008).

The outcome of many non-trophic interactions between pairs of species can be dependent upon the biotic and abiotic environmental conditions in which the interaction occurs (Thompson 1988). In particular, the occurrence of interspecific facilitation is often found to be more prevalent when conditions are marginal for at least one of the species involved, and some abiotic or biotic stress is ameliorated by one species to the benefit of the other (Bertness and Callaway 1994; Bronstein 1994; Hodge 2001). It has been suggested that plant pathogen-induced facilitation of insect herbivores is more likely to occur when the uninfected host-plant possesses high resistance or is in some way an inferior resource to the insects (Hodgson 1981). *Vicia faba* L. is considered one of the highest quality host plants for *A. pisum* due to its low aphid resistance, and it is possible that virusinfection could not improve (or degrade) the resource sufficiently to induce observable changes in aphid performance (Hodge and Powell 2008).

The aim of this investigation was to expand upon previous work by examining the response of *A. pisum* to PEMV infection of another commercially important host plant, *Pisum sativum* L. *A. pisum* performance can be affected by the age of the host plant, so the way in which the interaction between virus and vector can be modified was investigated by examining the age of the host and the severity of symptom development. In addition, the production of winged progeny by aphids on infected plants under isolated and crowded maternal conditions was examined, and settling preferences on whole plants and discs of infected leaf tissue was monitored.

## Materials and Methods

### General

Peas, *Pisum sativum* L. cv ‘Onward’ (Fabales: Fabaceae) were grown in an environment-controlled glasshouse with a 16:8 h day:night cycle, a minimum day-time temperature range of 15–18° C and a minimum night time temperature of 12–15° C. If required, light levels were supplemented with 400 W mercury fluorescent bulbs throughout the 16 h photophase. All plants were grown in compost with the addition of Perlite and Vermiculite (10:1:1 by volume) in 8 cm plastic pots and were watered as required with untreated water. Experiments were carried out in an insect growth facility with temperature maintained at 19±1° C, a relative humidity range of 50–80% and lighting provided by racks of six 65 W fluorescent tubes. *A. pisum* were restricted to plants by enclosing the plant in a perforated transparent plastic bag fastened around the pot using an elastic band.

The aphid used was clone JF01/29 of *A. pisum,* obtained from The Centre for Population Biology, Imperial College London (Silwood Park Campus), and was selected due to its high success rate in PEMV inoculation trials. Aphids were cultured at low density on seedlings of tic bean, *V. faba* var. *minor* L. (Fabales: Fabaceae) grown in pots of damp sand.

An isolate of PEMV was obtained from infected sweet peas, *Lathyrus odoratus* L. (Fabales: Fabaceae) collected in Ashford, Kent, UK, in June 2003, and maintained on *V. faba* by *A. pisum* transmission (Hodge and Powell 2008). Inoculation of experimental plants was performed by allowing *A. pisum* to feed on infected *V. faba* for three days to acquire the virus and then transferring three of them to each test plant for 24 hours. To account for any changes in the nutritional quality of *P. sativum* or induction of defences caused by aphid feeding controls, consisted of ‘sham-inoculations’, where aphids that had previously fed on uninfected beans were then allowed to feed on test plants for 24 hours. Infected peas were readily diagnosable after 12–14 days using visual symptoms: the correct visual diagnosis of infected (and control) plants was confirmed by ELISA on a sub-set (≊ 100) of plants from all experiments/treatments used throughout the study. Unless stated, plants were inoculated 11 days after sowing and used in assays 14 days post-inoculation.

### The effect of PEMV on survival and growth rate *of Acyrthosiphon pisum*

To assess the effect of plant infection and the severity of symptoms on survival and growth rate of *A. pisum,* nymphs (< 1 d) were weighed (Mettler, www.mt.com, Toledo MX5 micro-balance) and introduced onto plants at 0, 5, 10 and 15 days after inoculation, with a single aphid being allocated to each plant. The plants were bagged and placed in the insect growth room to allow the aphids to develop. The growth of aphids during these 5-day assays approximates an exponential curve (personal observation; see also van Emden 1969), modelled by the equation:

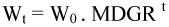

Where MDGR is the mean daily growth rate, t is the duration of the assay (in days), W_0_ is the initial weight and W_t_ the weight at time (t).

The MDGR of each *A. pisum* nymph can be estimated by:

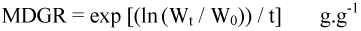



This mean daily growth rate parameter rather than final body weight was used in the statistical analysis, as it accounts for variation in the initial weights of aphids.

Because PEMV symptoms could not be seen in the inoculated plants in the 0- and 5-day post-inoculation treatments at the time the test aphids were introduced, these plants were returned to the glasshouse after the test aphid was removed to allow symptoms to develop. Only data from inoculated plants that ultimately expressed PEMV symptoms were included in the final data analysis (resulting in 50 to 88 viable replicates for each of the eight treatments).

### The effect of PEMV on the production of winged progeny by *Acyrthosiphon pisum*

In *A. pisum,* the switch to the production of winged alate progeny generally occurs due to maternal responses to cues from crowding and resource quality prior to each nymph being deposited onto the host plant (Müller et al. 2001). Thus, it was important to examine the effects of plant infection (and ingestion of modified sap, virus particles, *etc.*) separately from maternal crowding to see if plant infection alone could influence the production of alate forms. It was also desirable to examine whether plant infection by PEMV modified the effects of maternal crowding on alate production. Thus, two assays were performed to examine how exposure of adult *A. pisum* to PEMV-infected peas influenced the rate of offspring production and the proportion of these progeny that were alate: the first used single apterous founding adult *A. pisum* (10 d old) placed unrestricted onto pea plants so it had access to the entire plant under the perforated bag; the second assay used ten apterous founding *A. pisum* housed in a ‘clip-cage’ (2 cm diameter) attached to the plants so the aphids had access to the underside of a leaf. In both assays, the founding *A. pisum* were left on the test plants for 24 hours to reproduce. The progeny of these founding aphids were counted and then transferred to a tic bean seedling to develop for a further 10 days so the number of alate in each batch of nymphs could be established. Sixty replicates using individual founders and 50 with clip cages were set up for control and PEMV-infection treatments.

### Settling assays

Because one of the primary symptoms of PEMV is a reduction in plant growth, there are problems when examining the consequences of infection on aphid settlement. Although using equal-sized areas of infected and uninfected plant tissue is desirable - as it allows ease of comparison in terms of aphid settlement - some concession must be made by either using different ages of control and infected plants or cutting the plants in some way. If intact plants (or leaves) are used then the integrity of the system is maintained, but there will naturally be a discrepancy in the sizes of infected and uninfected hosts presented to *A. pisum*. As a compromise two methods were employed to examine the effect of PEMV infection on *A. pisum* settlement/arrestment: one assay using whole plants and a second using leaf discs.

In the first assay, the settling of alate aphids on whole plants was examined using a transparent Perspex wind tunnel (0.9 × 0.3 × 0.3 m) (see Du et al. 1996 for similar tunnel design). A fan and air filter system was fitted to one end of the tunnel, set to produce a horizontal airflow of 20 cm.s^-1^, with the exhaust air being vented from the room. Overhead lighting was provided by two 58 W linear fluorescent tubes that, with white paper placed on the roof of the tunnel, provided diffuse inside illumination of 25 µmol.m^-2^.s^-1^. Temperature, relative humidity and air pressure were measured using an electronic thermometer/hygrometer (Oregon Scientific, www.oregonscientific.com, model BAR913HG), and ranged between 19–21° C, 50–70% RH and 996–1006 mB during the assays. A control and PEMV-infected plant were placed 10 cm from the upwind end of the tunnel, so that the edges of the pots were 10 cm apart. For each trial, 20 post-teneral alates (11 d old) were released from a glass vial (50 × 25 mm diam.) positioned along the midline of the tunnel 20 cm downwind of the plants. After one hour, the alates settled on each plant were recorded. Twenty trials of the wind tunnel assay were carried out. The plants from each treatment were weighed (shoot fresh weight) to give an indication of the difference in size of control and infected plants.

To examine the settling preferences of *A. pisum* when presented with equal areas of infected and uninfected plant tissue, leaf discs (1 cm diam.) were cut from leaves using a stainless steel cork borer. The discs were placed adaxial side upwards in a plastic Petri dish (5 cm diameter) with a moistened filter paper (Whatman No 1) in the base. Two discs from infected leaves (0° and 180°) and two from a control plant (90° and 270°) were placed on the paper in an equidistant arrangement near to the edge of each Petri dish. Twelve *A. pisum* nymphs (< 2 d) were placed into the centre of each dish and the arenas were maintained in the insect growth room for four hours (before the leaf discs showed any visible signs of degradation), after which the distribution of settled aphids among the discs was recorded.

The leaf-disc assay was repeated in the absence of light to examine whether any preferences exhibited by the *A.pisum* were due to differences in visual cues. Arenas were set up as before, then placed into a black-lined light-proof box which was then placed into a darkened room. The distribution of the nymphs was again assessed after four hours, with arenas being removed from the light-proof box one at a time. One hundred arenas were set up for both the ‘light’ and ‘dark’ leaf disc assays.

### Statistical analysis

For the aphid performance experiments, survival and mean daily growth rate data were analyzed using generalized linear model (GLM) procedures, defining virus treatment and time since plant inoculation as factors. Survival was treated as a binary variable, utilizing a logit-link function in the GLM.

In the alate production experiments, χ^2^ tests were used to examine the association between plant infection and the presence of alate progeny. Because of the prevalence of zero counts of alates in some treatments, comparisons between the numbers of *A. pisum* (and proportion of alates) produced on healthy and infected plants were made using the non-parametric Mann-Whitney test.

For the preference assays, the difference between the numbers of *A. pisum* on the infected plants or discs and those on the controls was calculated for each replicate. The resulting set of differences was then tested against a median of zero using a non-parametric Wilcoxon test.

## Results

### The effect of PEMV on survival and growth rate of *Acyrthosiphon pisum*

**Figure 1.**
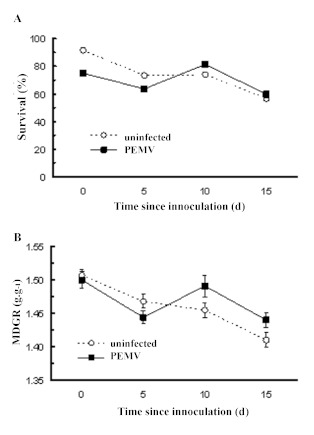
The effect of PEMV infection of peas on (A) five-day survivorship and (B) mean daily growth rate of *Acyrthosiphon pisum* (mean ± SE). High quality figures are available online.

The primary influence on *A. pisum* performance was the age of the plants at the time of aphid introduction and both performance measures exhibited similar negative trends with regard to plant age (Figure 1). In terms of aphid survival, on average those introduced onto the oldest plants (15 d post infection) had 30% lower survival than those introduced onto the youngest plants (χ^2^ = 7.3; P < 0.001 for 3 df). However, there was no effect of PEMV treatment on survival (Figure 1a; χ^2^ = 1.45; P > 0.2 for 1 df).

The *A. pisum* MDGR was similar on control and infected plants shortly after inoculation (Figure 1b; Days 0 and 5). However, when symptoms were more developed at the time of aphid introduction (Days 10 and 15) the average MDGR was significantly higher on the infected plants (Figure 1b; *age x PEMV,* F_3, 350_ = 3.1; P < 0.03). Although the improvement in daily growth rate on these highly-symptomatic plants was relatively small (∼ 3 %) this produced an average increase of ∼ 13% in the body weights of aphids feeding on infected plants over the course of the 5-day assay period.

### The effect of PEMV on the production of winged progeny by *Acyrthosiphon pisum*

With a single founding *A. pisum,* there were no significant differences in the number of offspring or the average proportion of alate when founders settled on control or infected plants (Table 1). Only 9 of the 120 founding aphids produced any winged offspring, and alates constituted only 3.3% of a total of 768 progeny.

Crowding the parent *A. pisum* in clip cages had the expected effect of inducing alate production, and 68% of cages contained alate progeny. On inspection, crowding inside the cages was so intense that many of them were not able to reach the leaf surface and, although ten founding aphids were used, the number of progeny retrieved after 24 hours was only six times that seen with individual founders. Although there was no difference in the total numbers of aphids produced on infected or control plants, the proportion of alates in the progeny of the aphids on PEMV-infected peas was significantly higher than on the controls (Table 1). Alates were produced in 82% of the cages attached to infected plants, compared to only 54% of the cages attached to controls (χ^2^ = 9.0, P < 0.005 for 1 df).

### Preference assays

**Figure 2.**
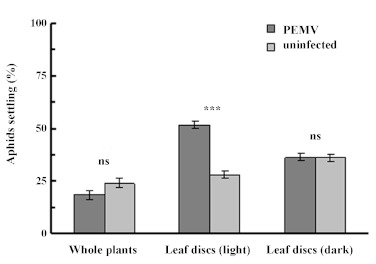
Proportion of *Acyrthosiphon pisum* settling on PEMV-infected or uninfected plants or leaf discs (mean ± SE; see Methods for details) (*** P < 0.001 ; ns — not significant). High quality figures are available online.

In the wind tunnel choice assays, the stunted PEMV-infected plants were on average only 60% the size of the control plants they were matched against (shoot fresh weight, 10.7 g v 6.4 g; t = 4.95, P < 0.001 for 16 df). However, this smaller size did not significantly affect the likelihood of alate *A. pisum* settling upon them (Figure 2; Wilcoxon statistic = 100, P > 0.10 for N = 16).

When an equal area of leaf tissue was presented to *A. pisum* in the form of leafdiscs, nymphs demonstrated a clear preference to settle on the infected leaf tissue, with almost twice as many nymphs being found on discs from infected leaves (52%) than the controls (28%) (Figure 2; Wilcoxon statistic = 3474, P < 0.001 for N = 91). However, this pattern was not observed when the leaf disc assay was performed in darkness, with equal numbers (36%) of nymphs being found on both categories of leaf disc (Figure 2; Wilcoxon statistic = 2314, P > 0.75 for N = 94).

**Table 1.**
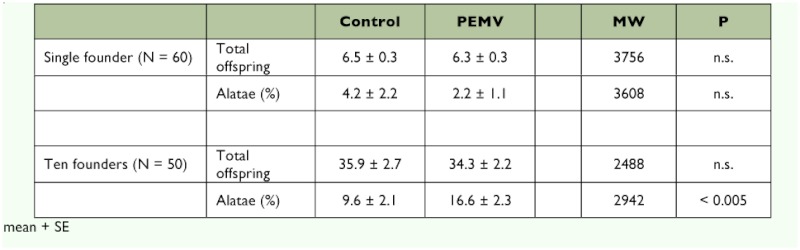
The effect of PEMV-infection of peas on the number of progeny and proportion of alatae produced by *Acyrthosiphon pisum* in 24 h.

## Discussion

The results from the settling assays substantiate previous findings where nymphs (and alatae and apterous adults) of *A. pisum* preferentially settled on leaf discs cut from PEMV-infected *V. faba* (Hodge and Powell 2008). *A. pisum* generally show a positive response towards yellow, in both plants and artificial ‘lures’, this colour in foliage representing physiological states (young leaves, senescence, disease, *etc.*) that constitute enhanced nutritional status (Kennedy et al. 1961; Moericke 1969; Dixon 1998). *A. pisum* have also been shown to respond positively towards volatiles released from virus-infected plants (Castle et al. 1998; Eigenbrode et al. 2002; Jimenez-Martinez et al. 2004), but the lack of settling preference observed under dark conditions strongly suggests they were responding to visual cues from the yellow/mottled colouring of the infected leaves (Macias and Mink 1969; Ajayi and Dewar 1983; Eckel and Lampert 1996; Fereres et al. 1999). Olfactory/surface chemical mechanisms may still have a secondary role in reinforcing visual signals and influence the likelihood of aphid arrestment (see Hardie et al. 1989; Blackmer and Cañas 2005). When whole plants were assessed, no difference in numbers of aphids settling on the control and infected plants was observed. However, the infected plants were only 60% of the size of the control plants (with no proportional decrease in settlement by the alate aphids), and the results suggest that adequate numbers of migratory *A. pisum* would still alight on infected plants (and acquire the virus) despite their smaller size.

Some prior investigations into plant virusaphid interactions have suggested that increased alate production on diseased plants is caused by physiological changes in the host plant, such as modification of nitrogen metabolism and changes in amino acid profile of the phloem sap (Gildow 1980, 1983; Fiebig et al. 2004). Poor nutrition seems an unlikely stimulus for alate production in the system used in this experiment, as the results of the aphid performance assays suggested that PEMV-infected peas were, if anything, superior hosts compared to control plants (*c.f.* Fiebig et al. 2004). Also, there was no increase in alate progeny when using a single founding *A. pisum,* indicating that infection of the plants *per se* (and any associated nutritional differences) did not directly induce production of winged forms. When multiple founding aphids were housed in clip cages the proportion of alate progeny on infected plants was almost double that observed on the controls. In terms of numbers of aphids, levels of crowding within the clip cages would be very similar in the control and PEMV-treated plants: the density of founding adults was equal, overall nymph production was not affected and any virus-induced increases in aphid size would only be slight within the short duration of the assay. Thus it appears that a combination of factors is required to produce the high numbers of alate progeny observed on the PEMV-infected plants, the effects of maternal crowding being somehow heightened when present in conjunction with host plant infection. The effects of crowding can be accentuated by higher contact rates resulting from increased restlessness of aphids, although this behavioural response was not examined explicitly (see Blua and Perring 1992b).

Although a single aphid growth parameter was used, it has been shown that individual growth rate and/or body weight is related to longer term performance measures such as reproductive output and population increase (*e.g.* Leather and Wellings 1981). The results of the *A. pisum* performance assays suggested that host plant quality was greatest on young pea seedlings, regardless of virus infection. Aphid growth was reduced on older plants, but the decline was less on those plants infected with PEMV. The growth response of aphids to plant infection by PEMV is not only conditional upon plant age and symptom development, but also on the host plant species as aphid growth was not modified on *V. faba* infected with this same strain of virus at similar levels of symptom development (Hodge and Powell 2008). These findings provide further indication that some complex insect vector/plant pathogen relationships could be regarded as mutualistic rather than commensal interactions when specific criteria are fulfilled (Castle and Berger 1993; Kluth et al. 2002; Belliure et al. 2005).

A caveat to the discussion of potential plantmediated effects on growth rate and alate production is that since PEMV is transmitted in a circulative manner the virions will also be present in the haemolymph and salivary glands of the aphid. Thus the direct effects of the virus inside the *A. pisum* cannot be separated from the indirect effects mediated via the symptomatic changes in the plant (see Ponsen 1969; Eliot et al. 2003; Belliure et al. 2005; Jiu et al. 2007). However, PEMV does not propagate within the *A. pisum* vector and we know of no mechanisms by which such direct facilitation of *A. pisum* by the virus could occur.

Although the model system used is of obvious agro-economic interest, there are few data available regarding the details of the ecological interactions that occur within this suite of virus-vector-host plant species, and the potential relevance of these interactions in terms of the epidemiology of the pathogen (see Jeger et al. 2004). From the results, it can be speculated that by exhibiting a positive settling response to PEMV-infected leaf tissue, *A. pisum* would subsequently experience an improved growth rate that, in combination with the smaller size of infected plants, would lead to more intense crowding and a higher propensity to produce alate offspring. These modifications in *A. pisum* behaviour and performance would individually or in combination — very likely result in enhanced dispersion and increased incidence of the virus within a stand of host plants. However, some caution is required when extrapolating from simplified laboratory models to field situations, and even some apparently positive responses such as the increased settling on infected tissue might have a debatable role in virus dispersion if, as a consequence, this behaviour decreases the likelihood of viruliferous *A. pisum* moving to an uninfected host (see McElhany et al. 1995; Sisterson 2008).

Whereas some aspects of plant virus-aphid interactions, such as the positive aphid settling response to yellowing infected leaves, appear to be quite general and widespread, others such as the modification of individual growth rate and production of alate progeny are more variable and dependent upon the species of aphid, host plant and pathogen that are considered (Hammond and Hardy 1988; Castle and Berger, 1993; Stout et al. 2006; Hodge and Powell 2008). In the PEMV-*A. pisum* systems examined, the distribution of *A. pisum* between healthy and infected plant tissues was dependent on the physical scale of the experimental arena and, to some extent, the age and morphology of the aphids considered (Hodge and Powell 2008; see also Macias and Mink 1969). The relative growth rate of *A. pisum* on infected plants was influenced by both the age of the host plants and the species of host plant considered (Markkula and Laurema 1964; Ellsbury et al. 1985) and the increase in alate progeny on infected plants was only observed when *A. pisum* were maintained at high density by simulated crowding using a clip cage. It becomes apparent that, as in many other investigations into the ecological interactions between species, the interactions that occur (or are inferred to have occurred) between a plant-virus and its aphid vector are dependent upon a combination of biological conditions and investigative protocol (Dodds 1988; Thompson 1988). Indeed, although variation in some experimental factors was examined in this experiment, variability in the outcome of the interactions or the intensity of any interspecific effects that might occur when utilizing different strains of PEMV, different cultivars of *P. sativum* and different clones/biotypes of *A. pisum* was not considered. Once it is accepted that some variability in the outcome of plant virus-insect herbivore interactions is actually the norm, then a more stochastic approach can be adopted to elucidate under what conditions, and in what manner, aphids are more or less likely to respond negatively or positively to pathogen infection of their host plant.

## Abbreviations

MDGR: mean daily growth rate
PEMV: *pea enation mosaic virus*
